# A Simple Vitamin Deficiency With Life-Threatening Complications: A Case of B12 Deficiency and Hyperhomocysteinemia-Induced Thrombosis

**DOI:** 10.7759/cureus.42908

**Published:** 2023-08-03

**Authors:** Laiba Khaliq, Kaiser F Kabir, Khin Pyai, Tarik Hadid, Benjamin Collins-Hamel

**Affiliations:** 1 Internal Medicine, Ascension Macomb Oakland, Warren, USA; 2 Internal Medicine, Michigan State University College of Osteopathic Medicine, Warren, USA; 3 Oncology, Ascension St. John Hospital and Medical Centre, Detroit, USA

**Keywords:** pernicious anemia, acute hemolytic anemia, intensive & critical care, hematology-oncology, obstructive shock, intrinsic factor, hyperhomocysteinemia, acute pulmonary embolism, vitamin b 12 deficiency

## Abstract

While macrocytic anemia is common in vitamin B12 deficiency, rarely, pancytopenia and hemolytic anemia can occur. Homocysteine levels are elevated in severe B12 deficiency, and this is linked to thrombus formation with potentially life-threatening complications. We present a patient with severe vitamin B12 deficiency complicated by hyperhomocysteinemia and obstructive shock from pulmonary embolism. A 56-year-old male with no medical history presented to the hospital with altered mentation. The patient’s family stated he was experiencing bilateral paresthesias of his lower extremities, progressive depression, anxiety, and insomnia. Initial vitals were blood pressure of 76/36, heart rate of 70 beats per minute, respiratory rate of 14, and temperature of 36.3 degrees Celsius. He was intubated due to severe encephalopathy. Relevant labs indicated severe macrocytic anemia, thrombocytopenia, decreased B12 levels, elevated methylmalonic acid, and elevated homocysteine. Imaging demonstrated a right common femoral vein thrombosis and subsegmental pulmonary emboli. Peripheral blood smear revealed schistocytes, anisopoikilocytosis, and decreased platelet count. The patient required fluid resuscitation, antibiotics, and multiple blood products. Vitamin B12 was administered intramuscularly, which improved the anemia. Esophagogastroduodenoscopy (EGD) demonstrated gastritis. Gastric and duodenal biopsies were negative for *Helicobacter pylori* and celiac disease. He was negative for intrinsic factor (IF) antibodies but had elevated gastrin levels. An intravenous unfractionated heparin infusion was started when the platelet count was above 50000. The patient was extubated after seven days. Heparin was transitioned to apixaban and an inferior vena cava (IVC) filter was placed. Hyperhomocysteinemia is a known pro-thrombotic factor that can lead to the development of venous thromboembolism. B12 malabsorption can stem from inflammatory bowel disease, celiac disease, gastritis, pancreatic insufficiency, gastrectomy, gastric bypass surgery, or antibodies to IF. While this case showed gastritis and negative IF antibodies, gastrin levels were elevated, indicating a mixed picture. This highlights the challenge of definitively diagnosing pernicious anemia as the cause of vitamin B12 deficiency. Vitamin B12 deficiency may lead to critical illness in which thromboembolism develops secondary to hyperhomocysteinemia.

## Introduction

Vitamin B12, also known as cobalamin, is a water-soluble vitamin that serves as a cofactor in DNA synthesis [1]. In Western countries, this vitamin deficiency is predominantly caused by malabsorption rather than dietary insufficiency [2]. Malabsorption can stem from inflammatory bowel disease, celiac disease, gastritis, pancreatic insufficiency, gastrectomy, gastric bypass surgery, or antibodies to IF [2]. While macrocytic anemia is a common manifestation of vitamin B12 deficiency, it is important to note that pancytopenia and hemolytic anemia can rarely develop. Homocysteine levels are elevated in B12 deficiency and this is linked to thrombus formation with eventual life-threatening complications [3]. We present a patient case with severe vitamin B12 deficiency secondary to likely pernicious anemia complicated by hyperhomocysteinemia and obstructive shock from pulmonary embolism. The patient’s condition improved with vitamin B12 supplementation. This presentation of severe vitamin B12 deficiency leading to critical illness is quite rare and reviews its investigation and successful management.

This case report was previously presented as a poster, at the state level, at the American College of Physicians (ACP) Michigan Chapter's Resident and Medical Student Day 2022 on May 13, 2022.

## Case presentation

A 56-year-old Caucasian male with no known past medical history presented to the hospital with altered mentation. The patient’s family stated he had been experiencing weakness and bilateral paresthesias of his lower extremities alongside worsening depression, anxiety, and insomnia. The patient was not a vegetarian, but his wife noted the patient had decreased appetite overall in the prior years. Initial vitals included a blood pressure of 76/36, heart rate of 70 beats per minute, respiratory rate of 14 breaths per minute, and temperature of 36.3 degrees Celsius. The patient was intubated due to severe encephalopathy. Labs indicated severe macrocytic anemia, thrombocytopenia, decreased B12 level with elevated methylmalonic acid, and elevated homocysteine. Table 1 includes initial labs and the results from investigating the etiology of anemia.

**Table 1:** Laboratory values These values represent the initial hospital laboratory findings alongside serological findings investigating pernicious anemia.

Table 1						
	Value	Reference Value				
Hemoglobin	1.4 g/dL	13.5-17.5g/dL				
Mean Corpuscular Volume (MCV)	137 fL	80-100 fL				
Reticulocyte	0.4%	0.70-1.80%				
Platelet	6 k/mcL	150-400 k/mcL				
International Normalized Ratio (INR)	3.5	-				
Aspartate Aminotransferase (AST)	644 unit/L	0-45 unit/L				
Alanine Transaminase (ALT)	1101 unit/L	0-45 unit/L				
Alkaline Phosphatase (ALP)	41 iUnits/L	20-130 iUnits/L				
Total Bilirubin	5.1 mg/dL	0-1.5 mg/dL				
Direct Bilirubin	2.8 mg/dL	0-0.4 mg/dL				
Indirect Bilirubin	2.3	0.2-0.8 mg/dL				
Albumin	4 gm/dL	3.5-5.0 gm/dL				
B12	<150 pg/mL	232-1,245 pg/mL				
Methylmalonic Acid	900 mcmol/L	0.00-0.40 mcmol/L				
Homocysteine Level	61 mcmol/L	0-14 mcmol/L				
Lactate Dehydrogenase	1555 iUnits/L	0-240 iUnits/L				
Haptoglobin	Undetectable	30-200 mg/dL				
Intrinsic Factor Antibodies	Negative	Negative				
Parietal Cell Antibodies	Negative	Negative				
Gastrin Level	818 pg/mL	0-100 pg/mL				
pH	6.9	7.35-7.45				
pCO2	41 mmHg	35-45 mmHg				
pO2	56 mmHg	75-100 mmHg				
HCO3	9 mmol/L	20-28 mmol/L				

**Figure 1:**
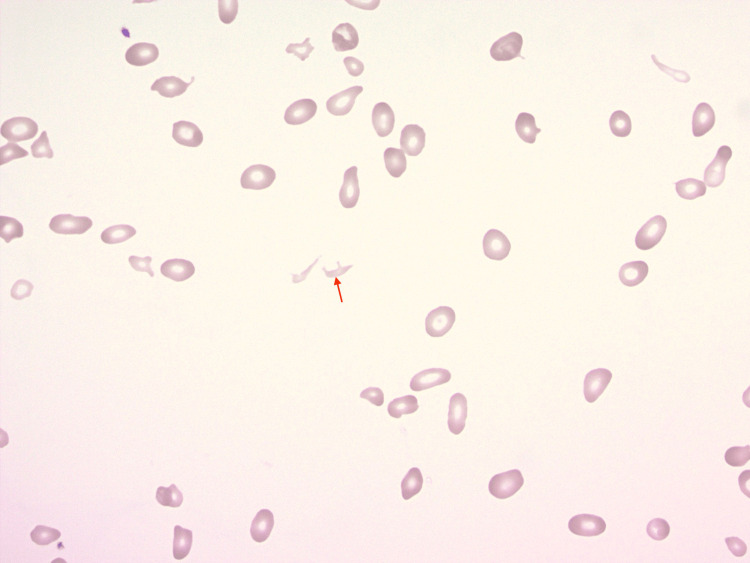
Peripheral blood smear A sample of the patient's blood smear with evidence of schistocytosis, anisocytosis, and poikilocytosis. The arrow indicates a schistocyte.

Imaging studies were performed to investigate suspected complications from the patient's symptoms. CT and ultrasound were positive for right common femoral vein thrombosis and subsegmental pulmonary emboli. Urinary retention and bladder distention was noted on imaging and a Foley catheter was placed after the patient was in the intensive care unit. Unfortunately, the Foley was placed into the prostatic urethra, leading to persistent hematuria. During the hospitalization, pancytopenia and hemolytic anemia also occurred. The patient's peripheral blood smear revealed schistocytosis, anisocytosis, poikilocytosis, and markedly decreased platelets without clumping (Figure 1).

The patient required fluid resuscitation, antibiotics, and multiple transfusions of packed red blood cells, fresh frozen plasma, and platelets. A supra-pubic catheter was placed to aid in hematuria resolution. Vitamin B12 1000 mcg was administered intramuscularly on the second day of hospitalization when elevated methylmalonic acid resulted; this was followed by a second intramuscular injection on the second day with the initiation of oral vitamin B12 1000 mcg via nasogastric tube, with significant improvement of anemia. Esophagoduodenoscopy was performed, which demonstrated gastritis. Gastric and duodenal biopsies were negative for *Helicobacter pylori *and celiac disease. Anticoagulation with intravenous unfractionated heparin infusion was initiated once hematuria resolved and platelet counts remained above 50,000. The patient was extubated after seven days. Heparin was transitioned to oral apixaban, and an inferior vena cava (IVC) filter was placed. Due to hospitalization-related debility, the patient completed an inpatient rehabilitation program before discharge. With appropriate vitamin B12 treatment, his levels increased and his thrombocytopenia, anemia, and coagulopathy improved (Table 2). After a total of 33 days in the hospital, the patient was discharged to a subacute rehabilitation facility. He exhibited clinical and hematologic recovery with complete resolution of thrombosis in the outpatient setting.

**Table 2:** Final blood count and hepatic function laboratory results Results from the final days of hospitalization show elevated B12 levels, resolution of anemia, thrombocytopenia, and coagulopathy.

	Value	Reference Value
Hemoglobin	8.5 g/dL	13.5-17.5 g/dL
Mean Corpuscular Volume (MCV)	96.8 fL	80-100 fL
Platelet	284 k/mcL	150-400 k/mcL
International Normalized Ratio (INR)	1.27	-
Aspartate Aminotransferase (AST)	26 unit/L	0-45 unit/L
Alanine Transaminase (ALT)	50 unit/L	0-45 unit/L
Alkaline Phosphatase (ALP)	iUnits/L	20-130 iUnits/L
Total Bilirubin	0.9 mg/dL	0-1.5 mg/dL
Direct Bilirubin	0.4 mg/dL	0-0.4 mg/dL
Albumin	3.3 gm/dL	3.5-5.0 gm/dL
B12	>2000 pg/mL	232-1,245 pg/mL

## Discussion

This case demonstrates the rare manifestations of severe vitamin B12 deficiency, including pancytopenia, hemolytic anemia, neuropsychiatric symptoms, and thrombosis due to hyperhomocysteinemia. As a consequence, this patient experienced a severe and prolonged hospital course. Commonly in practice, macrocytic anemia and hypersegmented neutrophils are observed as opposed to full pancytopenia [4]. The neuropathy of vitamin B12 is symmetric and caused by demyelination of the dorsal and lateral columns of the spinal cord, which was consistent with our patient’s symptoms [1,2]. Severe pancytopenia is observed in only 5% of patients and life-threatening signs of vitamin B12 occur in 10% of patients [5]. Hemolytic anemia is observed in 1.5% of patients with B12 deficiency [4]. Hemolysis may occur from intramedullary or extramedullary destruction of red blood cells but the exact mechanism is not well understood [4,6]. In vitro studies demonstrate that elevated levels of homocysteine can contribute to hemolysis as well [6]. This patient exhibited elevated LDH with undetectable haptoglobin levels indicating intramedullary hemolysis but still had many schistocytes on the peripheral smear.

Hyperhomocysteinemia is a known pro-thrombotic factor that can lead to the development of life-threatening venous thromboembolism, in addition to coronary artery disease and stroke [3,7]. A variety of mechanisms for hyperhomocysteinemia-induced thrombosis has been proposed. These mechanisms include relationships with promoting platelet aggregation, activating a variety of factors and the coagulation cascade, inducing endothelial injury, and increasing oxidative stress on the vasculature, which can activate proinflammatory biochemical pathways [7]. B12 deficiency takes approximately 5-10 years to develop due to large stores of the vitamin within the liver [8]. In most Western nations, gastrointestinal malabsorption is the most common cause of B12 deficiency [2]. Common causes of malabsorption include celiac disease, ulcerative colitis or Crohn's disease, gastritis, pancreatic insufficiency, gastric bypass surgery, or antibodies to IF [2]. Metformin, proton pump inhibitors, and H2 blockers are common medications linked to vitamin B12 deficiency also [1,2]. Pernicious anemia is responsible for about 75% of cases of B12 deficiency [9]. Screening for IF antibodies can be done, but in 30% of patients with pernicious anemia, antibody testing is negative [9]. Gastrin and pepsinogen levels are highly sensitive but their specificity is limited [10]. While this patient had gastritis and negative IF antibodies, gastrin levels were elevated, leading to a mixed picture. Given the severity of this patient's B12 deficiency as well as gastritis evident on endoscopy with his elevated gastrin levels, seronegative pernicious anemia as well as gastrinoma could have been possible. Unfortunately, inadequate parietal cell samples on biopsies make it difficult to make a conclusive statement on pernicious anemia. This exhibits the complexity of definitively diagnosing pernicious anemia as a cause of vitamin B12 deficiency, despite appropriate testing in the real world.

In addition to hematologists, our case also highlights the importance of other subspecialties, including gastroenterology and intensivists' role in recognizing the complications of severe vitamin B12 deficiency. The proper investigation of malabsorption includes endoscopic evaluation with a biopsy. The healthcare professionals in the intensive care unit have the closest contact with patients presenting with such severe disease and have a duty of properly following up on critical lab values and addressing them, in addition to evaluating the complications of obstructive shock and managing the mechanically ventilated patient. Appropriate follow-up with primary care is just as important upon hospital discharge, to continue monitoring the patient clinically as well can be crucial for evaluating vitamin B12 and homocysteine levels after treatment is initiated, oftentimes before they are able to see hematology. They also can augment the hematologist's ability to evaluate treatment effectiveness and to further educate patients so as to avoid further, subsequent, complications. Primary care also has a role in coordinating the care between different subspecialists, and in this case, ensuring continual recovery from prolonged hospitalization with appropriate outpatient physical therapy. Overall, severe B12 deficiency can lead to critical illness if thromboembolism develops, requiring an interprofessional approach to successfully investigate and manage.

## Conclusions

B12 deficiency can lead to pancytopenia, hemolytic anemia, psychiatric disturbances, cognitive slowing, and neuropathy. Common findings are macrocytic anemia, hypersegmented neutrophils, and elevated methylmalonic acid and homocysteine. However, pancytopenia is observed in only severe cases and hemolysis is a rare finding. Pernicious anemia is responsible for three-fourths of cases of vitamin B12 deficiency and appropriate workup must be initiated. IF antibodies may be present, but negative IF antibody results cannot truly rule out pernicious anemia as a cause. EGD with biopsy is necessary to investigate other absorption issues such as celiac disease. The diagnosis can be confirmed if hematologic manifestations of this vitamin deficiency are resolved with the administration of B12. Unfortunately, hyperhomocysteinemia is linked to thrombosis and, if not properly diagnosed and treated, can lead to life-threatening illness from thromboembolic disease.
